# Grieving Experiences of Parents with Children in End-of-Life Care—A Qualitative Review Protocol

**DOI:** 10.3390/nursrep12030041

**Published:** 2022-06-22

**Authors:** Maria Eduarda Correia, Tânia Melo, Joana Nobre

**Affiliations:** 1 Nursing Research, Innovation and Development Centre of Lisbon (CIDNUR), 1990-096 Lisboa, Portugal; 2 Health School, Polytechnic Institute of Portalegre, 7300-555 Portalegre, Portugal; joana.nobre@ipportalegre.pt; 3 Paediatric Hospital of the Centro Hospitalar e Universitário de Coimbra, 3000-602 Coimbra, Portugal; taniatmelo@esenfc.pt; 4 Nursing School of Coimbra, 3000-232 Coimbra, Portugal; 5 VALORIZA—Research Centre for Endogenous Resource Valorization, Polytechnic Institute of Portalegre, 7300-555 Portalegre, Portugal; 6 Nursing Research Unit for South and Islands (NURSE’IN), 2914-503 Setubal, Portugal

**Keywords:** grief, grieving, bereavement, parents, experience, end-of-life care, palliative care

## Abstract

Parents that accompany their children in end-of-life care until death go through a devastating, complex and vulnerable situation, which is experienced in a unique way and with individual needs, given the specificity of the palliative care setting in which it occurs. This qualitative review aims to identify and synthesize the best evidence available on the grieving experiences of parents with children in end-of-life care in a palliative care setting. This qualitative review protocol is according to the review method proposed by the Joanna Briggs Institute. The results will enable us to identify how parents of children in end-of-life care in a palliative care setting experience their grieving process. This review will broaden the horizon of understanding of the specificities of the grieving experience of parents who have accompanied their children in end-of-life care until their death in a palliative care setting and promote research in this context. The results of the review will also allow the construction of an “*end-of-life grieving script*”, with the aim of identifying one’s individuality in the grieving process. This protocol is registered at Open Science Framework.

## 1. Introduction

The technological development and scientific research in the last few decades of the last century have led to the development of all areas of health, changing the profile of patients and diseases. [1,2]. In addition, the area of child health and pediatrics has been brought undeniable progress, making the care provided increasingly developed and differentiated, leading to the increased survival of children with complex, limiting or life threatening chronic diseases [3,4].

In palliative care, far fewer deaths occur among the pediatric population than in adults [1]. However, children also die from life-threatening conditions that require end-of-life care in a pediatric palliative care setting [1,2,3].

Pediatric palliative care focuses on caring for children, preserving their dignity, promoting, and improving their quality of life by providing relief of suffering, using a holistic approach, managing symptoms, and providing psychosocial and spiritual care and support for the parents in their grief [1,2,3,5].

Parents’ experience throughout the disease process that culminates in end-of-life care and the death of their child puts them in a context of vulnerability that is inherent to the successive losses to which they are subjected. This context of vulnerability is packed with meanings that arise during the grieving process and are paramount in the search for meaning and explanation of the disease and death of the child, based on their beliefs and worldview [6,7,8,9].

The death of a child is a disturbing loss that leads to the parents’ suffering when they experience something they never expected to face, leading to profound consequences at an individual and family level because the relationship between parents and children is one of the most intense human relationships that exist [10,11,12,13].

Grief is the adaptive response to an experience of significant loss of affective attachment that leads to a complex process involving the physical, psychological, behavioural, spiritual, and socio-cultural dimensions of the human experience. In terms of emotional competence, the normal development of the grieving process leads to change in the attachment to the lost object and transfer to memories of the expressions of that same attachment, as a social event with the cultural customs and rituals that are part of the bereavement state [1,5].

However, the grieving experience is individual and unique. If it is not experienced adequately, it can become pathological and turn into a disorder that manifests as various permanent symptoms, causing incapacities at the family, professional and social levels [14,15,16].

Parents may also experience anticipatory grief, particularly parents of children in end-of-life care, as their role as caregivers does not let the physical and emotional burden that they experience become apparent when they project the loss of their child into the near future [17,18,19].

Recognizing the expressions and feelings of parents accompanying their children in end-of-life care, as well as their experience with unique specificities, is essential to anticipate the forms of adaptation that these parents adopt, which may favor or hinder the establishment of the grieving process [5,18].

To this end, interventions should be provided to the parents accompanying children in end-of-life care, valuing their parental and caregiver role, providing support, and ensuring that their needs are considered and based on knowledge and understanding of their experience [19].

An initial search carried out in JBI Evidence Synthesis, PubMed, CINAHL, and PROSPERO revealed that there are currently no qualitative reviews in preparation on the grieving experiences of parents accompanying their children in end-of-life care in a pediatric palliative care setting, highlighting gaps in the available evidence.

Therefore, it was decided that a qualitative review must be prepared, directed by the methodology proposed by the Joanna Briggs Institute [20], to identify and synthesize the best evidence available on the grieving experiences of parents of children in end-of-life care, following their death and in a palliative care setting.

More specifically, this review aims to answer the following question: what are the grieving experiences of parents accompanying their children in end-of-life care after their death in a palliative care setting?

## 2. Materials and Methods

This qualitative review will follow the method proposed by the Joanna Briggs Institute, aiming to identify and synthesize the best evidence available on the grieving experiences of parents accompanying their children until death in a pediatric palliative care setting. The criteria are defined based on the population, phenomenon of interest and context (PICo), identifying the inclusion and exclusion criteria [20]. The protocol has been registered at OSF (DOI: 10.17605/OSF.IO/9ZUNE).

### 2.1. Inclusion Criteria

#### 2.1.1. Population

Parents (mother and/or father) accompanying their children (aged 0 to 18 years) in end-of-life care due to an oncological or non-oncological disease until their death.

#### 2.1.2. Phenomenon of Interest

The grieving experiences of parents that accompany their children (aged 0 to 18 years) in end-of-life care, due to oncological and non-oncologic disease, following their death in a palliative care setting

#### 2.1.3. Context 

Pediatric palliative care, including assistance and/or care at home and/or in a hospital provided by pediatric palliative care units and/or teams, in any region of the world, to children in end-of-life care due to an oncological or a non-oncological disease.

#### 2.1.4. Types of Studies

The research will be conducted without a time limit and will include articles published in English, Portuguese and Spanish. This analysis will consider studies that focus on qualitative data, including, but not limited to, phenomenological, ethnographic or grounded theoretical studies on the mourning experiences of parents accompanying their children with a diagnosis of oncological or non-oncological disease in end-of-life care to death in the context of palliative care. 

### 2.2. Research Strategy

The search strategy will aim to locate both published and unpublished studies. To this end, an electronic search will be conducted in the following databases: MEDLINE (via PubMed), CINAHL (EBSCO), SciELO, Embase (Elsevier) and LILACS. The search for unpublished studies will include doctoral theses and master’s dissertations extracted from the following databases and gray literature in Open Access Scientific Repository of Portugal (RCAAP) and OpenGrey. 

The research strategy will be developed in the following three stages:

(1) The initial search is limited to MEDLINE (via PubMed) and CINAHL (via EBSCO) databases to analyze and group the most frequently used words found in the titles and abstracts and indexation terms of the articles.
(2) In a second stage, the identified keywords and index terms are combined and used in a single search performed in all included databases. The following is an example of a search strategy in the MEDLINE database (via PubMed), (Table 1).
(3) Finally, the bibliographic references of all identified articles and studies will be reviewed to search for and include additional studies.

The search results will be exported to the references’ manager Mendeley^®^ and duplicate articles will be removed. Based on the information provided in the title and abstract, two reviewers will independently assess the relevance of all the articles to this qualitative review. Possible disagreements between the reviewers will be resolved through discussion or with the inclusion of a third reviewer. The full text of all the studies that meet the inclusion criteria will be retrieved and read, and the analysis process described will be examined. Two independent reviewers will be analyzing the full text of the studies found to check if the previously defined inclusion criteria are met. The research findings will be reported in full in the final systematic review and presented in a flowchart of Preferred Reporting Items for Systematic Reviews and Meta-analyses (PRISMA) [21].

### 2.3. Methodological Quality Assessment

The JBI critical appraisal checklist for qualitative research [21] will be used to assess the methodological quality of the studies. Specifically, the selected studies’ reliability, relevance, and outcomes will be assessed. Two independent reviewers will carry out this assessment. If there are disagreements between them, a third reviewer will be used. Additionally, the authors of the primary studies may be contacted in case further clarification or missing data are required. The critical appraisal results will be reported in narrative and tabular form.

### 2.4. Data Extraction

Two independent reviewers will extract the data from the studies included in the review using the standardized JBI data extraction instrument and the JBI critical appraisal checklist for qualitative JBI research [21].

The data extracted will include specific details on the phenomenon of interest, participants, and outcomes of significance to the objective of this review. The primary authors of the studies may be contacted for further information if further clarification of the data is required. Two independent reviewers will extract the data. Any disagreement between the two reviewers will be discussed thoroughly. A third reviewer will be requested for final decisions if any disagreement persists.

### 2.5. Data Analysis and Presentation

Where possible, the results of the qualitative research will be grouped using the meta-aggregation approach and using the narrative method of presenting the results. 

## 3. Conclusions

The results of this review will synthesize the grieving experiences of parents accompanying their children in end-of-life care until their death in a palliative care setting and will be important to ensure that the needs of these parents are considered, based on an understanding of their experience.

## Figures and Tables

**Table 1:** Search strategy for MEDLINE (PubMed) conducted on 10 December 2021.

Search	Query	Records Retrieved
#1	(((parents [Title/Abstract]) OR (father [Title/Abstract])) OR (mother [Title/Abstract])) OR (parents [MeSH Terms])	345,575
#2	((((Mourning * [Title/Abstract]) OR (Bereave * [Title/Abstract])) OR (Grie * [Title/Abstract])) OR (Bereavement [MeSH Terms])) OR (Grief [MeSH Terms])	23,675
#3	((((((((((((“palliative care” [Title/Abstract] OR “palliative care” [Title/Abstract] OR “terminal care” [Title/Abstract] OR “palliative supportive care” [Title/Abstract] OR “supportive care” [Title/Abstract] OR “end of life care” [Title/Abstract] OR “hospice care” [Title/Abstract] OR “hospice nursing” [Title/Abstract] OR “palliative nursing” [Title/Abstract] OR “palliative treatment” [Title/Abstract] OR “palliative therapy” [Title/Abstract] OR “end of life” [Title/Abstract]) AND (“Hospice and Palliative Care Nursing” [MeSH Terms] OR “Palliative Care” [MeSH Terms] OR “Palliative Medicine” [MeSH Terms])	103,210
#4	#1 AND #2 AND #3	415
	Limited to Portuguese, English and Spanish results.	

## Data Availability

Not applicable.
